# Proteasome Inhibition Is an Effective Treatment Strategy for Microsporidia Infection in Honey Bees

**DOI:** 10.3390/biom11111600

**Published:** 2021-10-29

**Authors:** Emily M. Huntsman, Rachel M. Cho, Helen V. Kogan, Nora K. McNamara-Bordewick, Robert J. Tomko, Jonathan W. Snow

**Affiliations:** 1 Biology Department, Barnard College, New York, NY 10027, USA; emh2237@barnard.edu (E.M.H.); rmc2201@barnard.edu (R.M.C.); lena.vkogan@gmail.com (H.V.K.); noramcb@gmail.com (N.K.M.-B.); 2 Department of Biomedical Sciences, Florida State University College of Medicine, Tallahassee, FL 32306, USA; robert.tomko@med.fsu.edu

**Keywords:** *Nosema ceranae*, microsporidia, proteasome, therapeutic, pollination

## Abstract

The microsporidia *Nosema ceranae* is an obligate intracellular parasite that causes honey bee mortality and contributes to colony collapse. Fumagillin is presently the only pharmacological control for *N. ceranae* infections in honey bees. Resistance is already emerging, and alternative controls are critically needed. *Nosema* spp. exhibit increased sensitivity to heat shock, a common proteotoxic stress. Thus, we hypothesized that targeting the *Nosema* proteasome, the major protease removing misfolded proteins, might be effective against *N. ceranae* infections in honey bees. *Nosema* genome analysis and molecular modeling revealed an unexpectedly compact proteasome apparently lacking multiple canonical subunits, but with highly conserved proteolytic active sites expected to be receptive to FDA-approved proteasome inhibitors. Indeed, *N. ceranae* were strikingly sensitive to pharmacological disruption of proteasome function at doses that were well tolerated by honey bees. Thus, proteasome inhibition is a novel candidate treatment strategy for microsporidia infection in honey bees.

## 1. Introduction

The western honey bee, *Apis mellifera,* provides pollination services of critical importance to humans in both agricultural and ecological settings [1]. Honey bee colonies have suffered from increased mortality in recent years that is likely caused by a complex set of interacting stresses [2]. Among the environmental stressors linked to honey bee disease, there has been intensifying focus on the role of microbial attack on honey bee health [3]. The microsporidian species *Nosema ceranae* and *Nosema apis* can cause individual mortality in honey bees and have been implicated in colony collapse [4,5,6]. *N. apis* has been a well-appreciated pathogen of *A. mellifera* for a century. *N. ceranae*, which originated in the eastern honey bee, *Apis cerana*, was first observed in *A. mellifera* in the early 2000s and appears to have displaced *N. apis* in the western honey bee in many regions [5,6]. *N. ceranae*, an obligate intracellular parasite, is now one of the most common pathogens of the honey bee. Midgut infection by this unicellular eukaryote causes energetic stress, epithelial damage, and when untreated, death [7,8,9,10,11,12]. Furthermore, infection is associated with a number of physiological and behavioral changes that likely affect individual contribution to the colony [10,11,13,14,15].

In the United States, *N. ceranae* infection has traditionally been controlled by treatment with the drug fumagillin. However, its use is prohibited in Europe (reviewed in [16]) and its effectiveness in controlling *N. ceranae* at the colony level appears limited in scope and duration [17]. Equally troubling, high doses of this drug impact host cell function and evidence suggests that *N. ceranae* can evade suppression in some circumstances [18]. Critically, future availability of fumagillin is also uncertain due to production, safety, and market issues. Thus, efforts to find new treatment strategies are critical to protect honey bees from this parasite [16,19]. Many promising alternatives strategies for control of *N. ceranae* infection have now been pursued, including important studies exploring other small molecules, RNAi, probiotics, and various natural compounds ([20,21,22,23,24,25,26,27,28,29] and work prior to 2020 reviewed in [30]). Eukaryotic pathogens can be challenging to combat using chemical antimicrobials because of the phylogenetic closeness with their hosts, and microsporidia are no exception. However, comparative genomics indicates that microsporidia have lost many of the cellular processes and pathways found in free-living eukaryotes [31], perhaps suggesting a loss of the redundancy and flexibility that often allows organisms to withstand cellular stresses. In support of this, fumagillin, as a methionine aminopeptidase 2 inhibitor, works by interfering with protein synthesis, thereby disrupting protein homeostasis, or the homeostasis of protein synthesis, folding, function, and degradation. In addition to sensitivity to fumagillin, *N. ceranae* exhibit vulnerability to other proteotoxic stresses, including thermal stress, which we previously confirmed in parallel with characterization of the response to heat shock in this species [32], and tRNA synthetase inhibition [33]. The regulated clearance of proteins is another aspect of protein homeostasis that is critical to the functionality of the proteome [34,35]. This process is mediated by a number of pathways including degradation by the ubiquitin–proteasome system (UPS) [36].

The UPS is a complex system that targets proteins for regulated degradation in an energy-dependent manner. Proteins to be destroyed are typically first covalently modified with a chain of the small protein ubiquitin, which serves as a signal for recognition by the proteasome. The proteasome is a large, multi-subunit, ATP-dependent protease complex that cleaves substrates into short peptides and recycles the polyubiquitin targeting signal. In addition to the clearance of damaged or misfolded proteins, the proteasome also serves an important regulatory function by destroying signaling proteins involved in various cellular processes.

The archetypal 26S proteasome is made up of a barrel-shaped core 20S proteolytic complex known as the core particle (CP) and one or two 19S regulatory particles (RP) that cap the barrel ends. The RP can be biochemically divided into two subcomplexes, the lid and the base [37,38]. The base contains six AAA+ family ATPase subunits, Rpt1-6, and three non-ATPase subunits, Rpn1, Rpn2, and Rpn13. The lid consists of nine non-ATPase subunits, Rpn3, Rpn5-9, Rpn11, Rpn12, and Rpn15/Sem1. One additional subunit, Rpn10, associates with the otherwise fully assembled RP and contacts both the lid and the base.

The RP is responsible for initial substrate capture via the ubiquitin-binding activities of Rpn1, Rpn10, or Rpn13. Once the substrate is captured, the polyubiquitin chain is removed by an intrinsic deubiquitinating activity within the Rpn11 lid subunit, and the substrate is unfolded via ATP-dependent mechanical motions of the ATPases Rpt1-6. The unfolding process also translocates the substrate into the center of the CP barrel for proteolysis. The CP consists of four axially stacked heteroheptameric rings of 7 α subunits or 7 β subunits. The α rings are responsible for interfacing with the RP and controlling access to the peptidase sites, whereas the proteolytic activity is housed within the β rings and is mediated by three of the β subunits (β1, β2, and β5). The β1, β2, and β5 subunits are referred to as caspase-like, trypsin-like, and chymotrypsin-like, based on their cleavage preferences [36].

As a sensitivity of microsporidia to UPS disruption might also be expected due to their compacted genomes, we examined genes associated with the UPS in *N. ceranae* and found that this species has a degenerate proteasome apparently lacking multiple components observed in most eukaryotes. However, based on sequence analysis and structural modeling of the key catalytic subunit, β5, proteasome inhibitors were predicted to disrupt function. In agreement with this supposition, we observed a striking sensitivity of *N. ceranae* to pharmacological disruption of proteasome function without a concomitant reduction in honey bee survival, suggesting the possibility of exploring proteasome inhibitors as novel anti-Nosema chemical agents.

## 2. Materials and Methods

### 2.1. Honey Bee Colonies and Caging Experiments

Honey bees were collected from outbred colonies in New York, New York consisting of a typical mix of *Apis mellifera* subspecies found in North America, at different times during the months of April–October. Source colonies were visually inspected for symptoms of common bacterial, fungal, and viral diseases of honey bees. For caging experiments, bees were collected from the landing board or newly emerged bees were collected after hatching from a capped brood frame overnight in an incubator at 35 °C in the presence of PseudoQueen (Contech, Victoria, British Columbia, Canada) as a source of Queen Mandibular Pheromone (QMP). Approximately 20 landing board bees or 30 newly eclosed bees were placed in each 4.8 × 3.4 × 8.4 inch acrylic cage with sliding door machined at Carelton Labs, Columbia University. For cages containing newly eclosed bees, approximately 4 foragers from the same source colony (marked with a spot of paint (Testors, Vernon Hills, IL, USA) were added to each cage. Caged bees were maintained in incubators at 35 °C (unless otherwise stated) in the presence of PseudoQueen (Contech, Victoria, BC, Canada) as a source of Queen Mandibular Pheromone (QMP).

### 2.2. Nosema ceranae Spore Isolation and Quantification

*N. ceranae* spores were obtained from infected individuals for use in infection studies [8,39]. In addition, an isolate was obtained from this colony and serially passaged through bees as performed previously [40]. Spores from these bees were used in most experiments. The species of Nosema used for infection was verified by qPCR. To isolate spores, midguts from infected or uninfected bees were individually crushed in 0.5 mL H20 and spore number was assessed by light microscopy. Midguts were washed 3 times with water and resuspended in 33% sucrose solution at a concentration of 1 × 10^6^ spores per mL for landing board bees or 5 × 10^6^ spores per mL for newly eclosed bees.

### 2.3. Nosema ceranae Infections and Chemical Treatments

For experiments with landing board bees, caged bees were allowed to consume food containing spores ad libitum for 24 h before food was replaced with sucrose solution alone. Bees in the uninfected group always received sucrose solution containing a midgut from an uninfected bee processed in the same way as the midgut containing spores. For experiments with newly emerged bees, caged bees were fed sucrose solution and supplied with a ~5 g pollen substitute patty (1:1 mix of BeePro and sucrose solution). On day 2 post-eclosion, *N. ceranae* spores (5 × 10^6^/mL) were fed to bees in sucrose solution ad libitum [41] for 48 h. At 3 days post infection, honey bees in individual cages (landing board bees and newly eclosed bees) were fed sucrose solution containing one of the pharmacologic agents (Supplemental Table S4) or vehicle control alone (DMSO) at the indicated doses. After 4 days of drug feeding, honey bee midguts were dissected, crushed in 0.5 mL water, and the number of mature spores counted by light microscopy as previously described [41]. In parallel, qPCR was used to determine relative amount of *N. ceranae* genome equivalents versus host genome equivalents.

For survival experiments, newly eclosed bees were caged and fed as above, but were left uninfected. Starting on 4 days post eclosion, bees were switched to sucrose solution containing one of the pharmacologic agents (Supplemental Table S4) or vehicle control alone (DMSO) at the indicated dose for 10 days while survival was assessed.

### 2.4. DNA Extraction and qPCR

DNA extraction was performed using a modified Smash and Grab DNA Miniprep protocol as described previously [42]. The resulting DNA was used as a template for qPCR to determine the levels infection for *Nosema* sp. using primer sequences for the *N. apis 16S* gene, the *N. ceranae β-actin* gene, and the honey bee *ATP5a* gene [42]. Reactions were performed with PowerUp SYBR Green Master Mix (Applied Biosystems, Foster City, CA, USA) in a LightCycler 480 thermal-cycler (Roche, Branchburg, NJ, USA). The difference between the threshold cycle (Ct) number for honey bee *ATP5a* and that of the *Nosema* sp. gene of interest was used to calculate the relative infection level using the ΔΔC_T_ method. A sample was considered negative for a specific *Nosema* species if it did not amplify any product by 35 cycles and zero was entered as the value in these cases.

### 2.5. Ortholog Screening of the N. ceranae and Other Microsporidian Genomes

The KEGG (Kyoto Encyclopedia of Genes and Genomes) database was used as a guide for comparing pathways between species [43]. In addition to looking at proteasome pathway gene candidates predicted by this database (nce03050), pathway genes from *S. cerevisiae* (sce03050) were used to find orthologs in the *N. ceranae* genome as well as other available microsporidian genomes using the BLAST family of search functions (www.ncbi.nlm.nih.gov (accessed on 12 August 2020)) as described previously [44]. Coding sequences from human or other eukaryotes were also used as queries to further support the absence of selected genes from microsporidian genomes.

### 2.6. CP α Subunit Ortholog Assignment and N. ceranae α Ring Homology Model Generation

After unsuccessful assignment of α subunit orthologs by pairwise comparison to human and *S. cerevisiae* genomes using BLAST, a protein threading approach guided by secondary structure and residue exposure was utilized. Specifically, the CPHmodels 3.2 server (http://www.cbs.dtu.dk/services/CPHmodels/ (accessed on 12 August 2020)) was used to identify best templates and to generate PDB models of each *N. ceranae* α subunit. The best-fit model in each case confidently identified a specific α subunit (E: <10^−20^), except for XP_024331661.1, which yielded a low-confidence fit to *B. taurus* α2 from PDB ID 1IRU. Remote homology modeling using the *T. acidophilum* α subunit yielded a high-confidence homology model for this protein. Considering the high-confidence identification of homologs for the other six subunits (α2–α7) using this approach, we tentatively assigned XP_024331661.1 as α1.

We next assembled a homology model of the α ring by superimposing each subunit onto its ortholog in the *S. cerevisiae* CP crystal structure (PDB ID = 1RYP) using Pymol 1.3r1 (Schrödinger, Inc. New York, NY, USA.This yielded a model with little to no apparent steric conflict. In the model, a lysine residue was positioned appropriately in each α-α interface for salt bridging with Rpt subunit C-termini as reported for the Archaeal, yeast, and human proteasomes (note that α7 contains a conservative arginine substitution at this position instead of a lysine). Importantly, the pocket formed by α7 and our tentative α1 subunit lacked a lysine residue contributed by α1 for salt bridging, which is a conserved feature of the CP from other eukaryotes. Finally, assuming the arrangement of Rpt subunits and the register of the RP relative to the CP is conserved in the *N. ceranae* RP, the resultant model would align the lone Rpt tail that lacks a Hb-Y-X motif, Rpt4, with the lysineless α7-α1 pocket as observed in other eukaryotic CPs. These observations further supported the assignment of XP_024331661.1 as *N. ceranae* α1.

### 2.7. Molecular Modeling of the N. ceranae β5-Ixazomib Complex

A homology model of *N. ceranae* β5 was generated using CPHmodels 3.2 as above. The model was then superimposed on chain b of the crystal structure of ixazomib bound to the human CP (PDB ID = 5LF7) using the “align” command in Pymol, yielding a fit with RMSD = 1.567 Å. Steric clash was assessed via visual inspection and conservation of salt bridging or hydrogen bonding was inferred from inter-atom spacing of ≤4 Å.

### 2.8. Statistical Analysis

Data is presented as means ± SEM shown. For two groups, data was compared using unpaired t-tests with Welch’s correction when values fit normal distributions or Mann–Whitney U nonparametric tests when they did not fit normal distributions. Normality was assessed using Shapiro–Wilk tests. When more than two groups were being compared, data was compared using one-way ANOVA with Tukey’s multiple comparison test when values fit normal distributions or a Kruskall–Wallis test when they did not. For survival analysis, treated versus untreated groups were compared using the Gehan–Breslow–Wilcoxon test.

## 3. Results

### 3.1. N. ceranae Lack Obvious Orthologs of Several Proteasome Subunits, Proteasomal Assembly Chaperones, and the Proteasome Regulatory Transcription Factor Rpn4

Using *Saccharomyces cerevisiae* proteins as queries [45], we searched for proteasome components in a number of disparate microsporidia genomes, including *N. ceranae*, *N. apis*, *Encephalitozoon hellem, Nematocida* *displodere,* and *Mitosporidium daphnia* (which represents an early diverging microsporidian species that does not demonstrate the genome compaction observed in other microsporidia [46]). We also examined proteasome components in *Rozella allomycis,* which is a member of the *Cryptomycota* group that is closely related to microsporidia [46], and three other fungal species, *Candida albicans*, *Aspergillus fumigatus*, and *Schizosaccharomyces pombe*.

**Figure 1:**
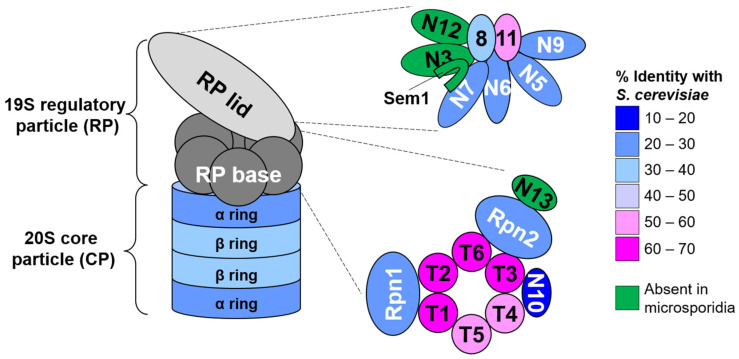
Ultrastructure and conservation of the *N. ceranae* 26S proteasome. Cartoon model of the canonical 26S proteasome. The 19S regulatory particle (RP) consists of lid and base subcomplexes. Detailed subunit compositions are shown to the right. The 20S core particle (CP) consists of four stacked heteroheptameric rings of α and β subunits. The coloring of the subunits indicates their sequence identity with their respective *S. cerevisiae* orthologs. For the CP, the average sequence identity of the seven subunits is displayed. Subunits shown in green appear to be absent from *N. ceranae* and all other microsporidia examined.

First, we searched for proteins making up the RP. Although obvious homologs of most RP subunits could be identified in each species, we were unable to find orthologs of the lid subunits Rpn3, Rpn12, and Rpn15/Sem1 or the base subunit and ubiquitin receptor Rpn13 in any of the species examined (Figure 1, Supplemental Table S1 and Video S1). Use of other fungal or mammalian orthologs as queries also failed to yield any homologs. Alignment of RP subunit protein sequences from *N. ceranae* with those of *S. cerevisiae*, whose proteasome has been visualized at near-atomic resolution [47,48,49,50,51,52,53], revealed that the enzymatic subunits of the RP (Rpn11 and the six Rpt subunits) have the highest overall sequence identity to that of *S. cerevisiae*, particularly within the catalytic domains (Figure 1; Supplemental Video S1). A notable feature with respect both to lid and base subunits was the truncation of selected N- and C-termini of the *N. ceranae* subunits. Prominent among these truncations were the N-termini of lid subunits Rpn5, Rpn6, and Rpn7. In yeast and other eukaryotes, the N-termini of these subunits make critical contacts with the CP and/or the ATPase ring of the base [54,55,56,57], raising the possibility that communication between these subcomplexes may be altered in microsporidia. Alterations in the lengths or sequences of lid subunits that create a key helical bundle critical for assembly and stability of the lid were also evident; these likely evolved to accommodate the absence of Rpn3, Rpn12, and Rpn15/Sem1, which have key roles in the assembly and stability of the lid in yeast [58,59,60,61].

Within the base, the N-termini of several Rpt subunits were truncated somewhat compared to their *S. cerevisiae* orthologs, but the significance of this is not immediately evident. Overall, the non-ATPase subunits Rpn1 and Rpn2 displayed the least sequence conservation with that of *S. cerevisiae* (~22% identity each vs. ~60% for each Rpt subunit). Further, key functional regions of both subunits were absent. These included a number of residues within Rpn1 responsible for ubiquitin binding in the *S. cerevisiae* subunit that were not obviously conserved [62,63] (Supplemental Figure S1A), and a large C- terminal truncation that removes the full Rpn13-binding region of Rpn2 [64,65,66] (Supplemental Figure S1B). This is consistent with the absence of an obvious Rpn13 ortholog in microsporidia, and together with the relatively poor conservation of known ubiquitin-binding residues on Rpn1 raises the possibility that Rpn10 may be the sole intrinsic ubiquitin receptor in this family of organisms.

With respect to the CP, all microsporidia species examined encoded at least 7 apparent α subunits and 7 apparent β subunits, consistent with other eukaryotes studied to date (Figure 1, Supplemental Table S2). Individual *N. ceranae* β subunits could be confidently matched to their orthologs using simple sequence homology searches. As is the case in *S. cerevisiae*, five of the seven β subunits appear to encode N-terminal propeptides that are anticipated to be cleaved during proteasome biogenesis, and the catalytic residues of the β1, β2, and β5 orthologs are readily identified (not shown and see below).

In contrast, individual α subunits could not be confidently matched to a particular ortholog upon pairwise sequence comparisons to α subunits of *S. cerevisiae* or humans. However, a structure-based homology search in combination with analysis of known distinguishing features of the yeast/human α ring permitted a reasonably confident identification of orthologs, and generation of a homology model of the *N. ceranae* α-ring built on the *S. cerevisiae* structure [67] (Supplemental Figure S2). Confidence in this model is supported by the presence of a conserved “pocket” lysine at the interfaces of all α subunit pairs except *N. ceranae* α1 and α7 (Supplemental Figure S2 and Table S3), as is the case in the human and yeast α rings. This lysine forms a salt bridge with the C-terminal carboxylate and/or a conserved tyrosine or phenylalanine residue of a particular Rpt subunit in the RP [68,69]. This tyrosine or phenylalanine is part of a so-called Hb-Y-X motif (where Hb is a hydrophobic residue and X is any amino acid), and is present in Rpt1, Rpt2, Rpt3, Rpt5, and Rpt6 in other eukaryotes. Docking of these Hb-Y-X motifs in turn opens a proteinaceous gate in the α ring, providing the substrate access to the CP peptidase sites for proteolysis [47,68,70]. Hb-Y-X motifs are similarly present at the C-termini of *N. ceranae* Rpt1, Rpt2, Rpt3, Rpt5, and Rpt6 (Rpt6 contains a hydrophobic residue but has a methionine in place of the tyrosine or phenylalanine). This suggests that a similar mechanism for gating is likely employed. However, the conserved YDR motif found in the N-termini of the α subunits of other eukaryotes that forms the gate of the CP [47,71] was notably absent in all seven *N. ceranae* subunits, suggesting an alternative structure of the proteinaceous gate.

We also searched for orthologs of the 11 known dedicated proteasome assembly chaperones, the proteasome modulator Blm10, and the proteasome-associated deubiquitinating enzyme Ubp6 (Supplemental Table S4). However, obvious orthologs for each of these, with the possible exception of Ubp6, were absent in virtually all microsporidia examined. These absences suggest potentially altered assembly and regulation of the proteasome in microsporidia, although it should be noted that the sequence conservation of some assembly chaperones is very weak even among other eukaryotes [72].

The biogenesis and function of the proteasome is highly regulated [73,74] via both transcriptional and post-translational mechanisms that control both the amount and activity of proteasome components [75]. The steady-state levels of the proteasome are tightly controlled in yeast [76,77], worms [78], flies [79], and mammals [80], although the mechanisms are distinct. In *S. cerevisiae*, expression of proteasome components is regulated by the transcription factor Rpn4, which binds to a 9 bp upstream activating sequence termed the proteasome-associated control element (PACE). Degradation of Rpn4 by the proteasome [76,77] in turn serves as a direct method for conveying proteasome capacity information to the regulation of proteasome component gene expression by suppressing new proteasome subunit synthesis. Examination of the genomes of *N. ceranae* and other microsporidia revealed that these species do not possess obvious Rpn4 homologs, suggesting an altered mode of regulation of proteasome gene expression in these species. We also found no instances of the canonical PACE (GGTGGCAAA) in any of the CP or RP genes of *N. ceranae* (gene body +/− 500 bp of additional sequence examined, data not shown). It is important to note that while the Rpn4/PACE system is highly conserved in most fungi, it is not operative in some fungi, such as *S. pombe* [81], suggesting that novel mechanisms regulating proteasome component expression in fungi exist that could be important for microsporidia.

### 3.2. Proteasome Inhibition Controls Existing Infections by N. ceranae in Experimentally and Naturally Infected Bees

**Figure 2:**
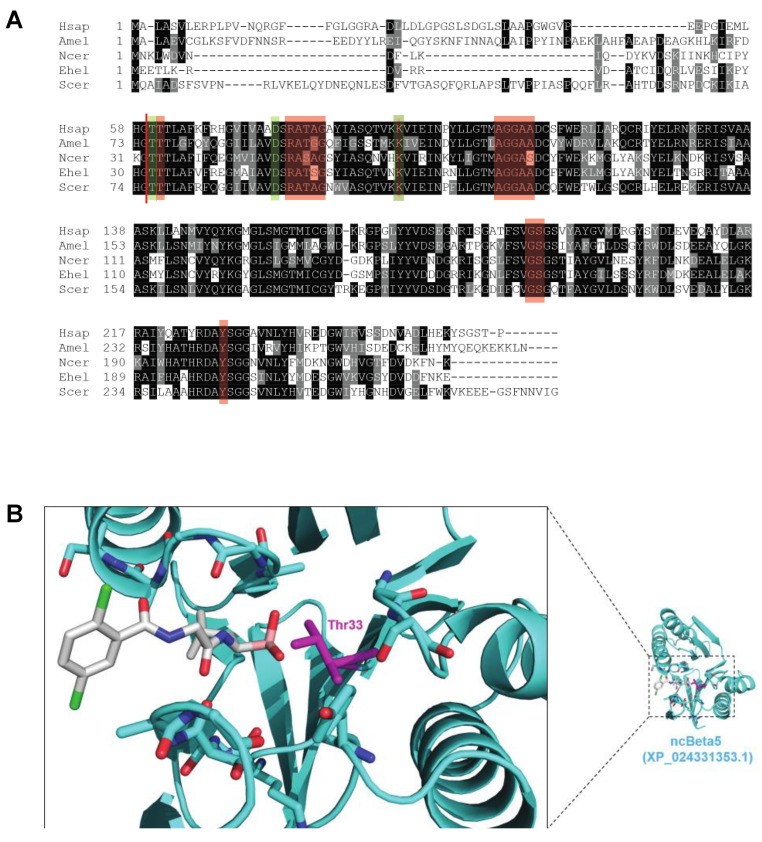
Conservation of key sequence and structural features of the β5 active site. (**A**) Sequence alignment of the 20S proteasome β5 subunit from *H. sapiens* (NP_002788.1), *A. mellifera* (XP_394680.3), *N. ceranae* (XP_024331353.1), *E. hellem* (XP_003887862.1), and *S. cerevisiae* (NP_015428.1). Amino acids from the catalytic triad (T1, D17, and K33 numbered from processed *H. sapiens* protein [83]) are boxed in green and amino acids critical for binding proteasome inhibitors (T2, R19, A20, T21, A22, G23, K33, A46, G47, G48, A49, A50, G129, S130, Y169, numbered from processed *H. sapiens* protein [82]) are boxed in red. The processing site for generating the mature protein is denoted with a red line. (**B**) Homology model of *N. ceranae* β5 bound to ixazomib. The β5 subunit is shown in ribbon mode. Residues forming electrostatic interactions with ixazomib in the crystal structure of ixazomib with the human CP are shown in stick mode, with the catalytic threonine colored magenta. Ixazomib is shown in stick mode with the boronate boron atom colored salmon.

We wished to determine how proteasome inhibition would impact *N. ceranae* infection in honey bees. Many proteasome inhibitors are known to target the active site in 20S proteasome subunit β5 and its interface with 20S proteasome subunit β6 [82]. We first aligned the 20S proteasome subunit β5 homologs from *H. sapiens* (NP_002788.1), *A. mellifera* (XP_394680.3), *N. ceranae* (XP_024331353.1), *E. hellem* (XP_003887862.1), and *S. cerevisiae* (NP_015428.1). We found all three of the amino acids thought to be in catalytic triad (T1, D17, and K33 numbered from processed *H. sapiens* protein [83]) to be conserved. We also found a high degree of conservation for the amino acids critical for binding proteasome inhibitors (T2, R19, A20, T21, A22, G23, K33, A46, G47, G48, A49, A50, G129, S130, Y169, numbered from processed *H. sapiens* protein [82]) (Figure 2A). Other microsporidial β5 proteins possess similar levels of conservation (Supplemental Figure S3). We exploited the high sequence identity between β5 orthologs to produce a molecular model of the β5 subunit from *N. ceranae* bound to ixazomib, a modified peptide boronic acid that binds the β5 site of the 20 S proteasome and inhibits proteasome activity [84] (Figure 2B). In this model, no steric conflict between β5 and ixazomib was apparent, and many of the salt bridges and hydrogen bonds that stabilize the drug in the β5 active site were evident. Thus, we predicted that ixazomib, and likely many proteasome inhibitors, would bind to the β5 proteins in microsporidia and disrupt proteasome function.

**Figure 3:**
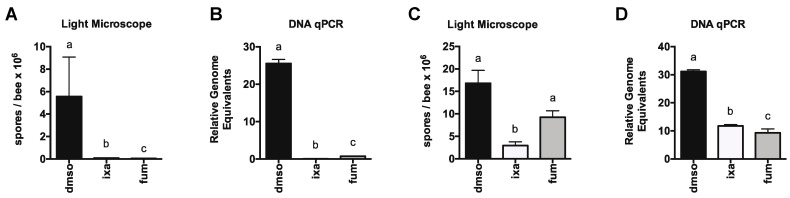
Proteasome inhibition reduces *N. ceranae* infection intensity in experimentally or naturally infected honey bees. *N. ceranae* levels in midguts as determined by spore count using light microscopy (**A**) or by qPCR (**B**) 8 days post infection in uninfected or infected landing board bees fed sucrose solution containing DMSO or ixazomib for the final 4 days. *N. ceranae* levels as determined by spore count using light microscopy (**C**) or by qPCR (**D**) in individual landing board bees from an infected colony captured and fed sucrose solution containing DMSO or ixazomib for 2 days, a ≠ b ≠ c, *p* < 0.05.

Toward this goal, we first tested the ability of ixazomib to reduce *N. ceranae* infection in honey bees. After experimentally infecting bees collected from the landing board of an uninfected colony, we fed bees sucrose solution containing vehicle DMSO, 40 µM ixazomib, or 40 µM fumagillin for 4 days starting on 4 days post infection. On day 8 post infection, we then measured spore levels using light microscopy and the amounts of *N. ceranae β-actin* gene relative to honey bee *ATP5a* gene by qPCR (which allows measurement of all life stages of *N. ceranae* unlike spore counting) to determine the effects of proteasome inhibition on *N. ceranae* infection intensity. We found that feeding infected bees ixazomib for 96 h resulted in a dramatic reduction in infection intensity in infected bees by both measures (Figure 3A,B). Using bees from a highly infected colony (prevalence >90% infected), we fed bees sucrose solution containing vehicle DMSO, 40 µM ixazomib, or 40 µM fumagillin and observed a striking reduction in *N. ceranae* infection by both spore-counting and DNA analysis (Figure 3C,D).

These results justified further testing of ixazomib and other proteasome inhibitors as possible anti-Nosema agents. Significant effort has been directed towards finding novel strategies for targeting the proteasome [85,86,87], providing a number of pharmacologic agents with varying efficacy, bioavailability, and selectivity. To standardize experiments by using age-matched bees and to allow for longer treatment periods, we used newly eclosed bees and tested the effects of a number of commercially available proteasome inhibitors, including ixazomib (MLN2238), ixazomib citrate (MLN9708), oprozomib (ONX 0912), dexazomib, carfilzomib, bortezomib, epoxomicin, HMB-Val-Ser-Leu-VE, MG262, and the two stereoisomers of MG132 (S and R) as well as fumagillin (Supplemental Table S5). On day 2 post-eclosion, *N. ceranae* spores (5 × 10^6^/mL) were fed to bees in sucrose solution ad libitum [41] for 48 h. At 3 days post infection, honey bees in individual cages were fed sucrose solution containing one of the pharmacologic agents at 40 µM or vehicle control alone. For each trial, we individually tested two novel compounds simultaneously with an untreated group, a fumagillin treated group, and an ixazomib-treated group. After 4 days of drug feeding, honey bee midguts were dissected, and infection levels were assessed by spore counting and qPCR (Supplemental Figure S4). We observed reductions in infection level by relative genome equivalents for all tested proteasome inhibitors except HMB-Val-Ser-Leu-VE (Supplemental Figure S5 and Table S6). There were varying levels of impact on *N. ceranae* infection with ixazomib and its citrate salt being the most effective at reducing infection levels.

**Figure 4:**
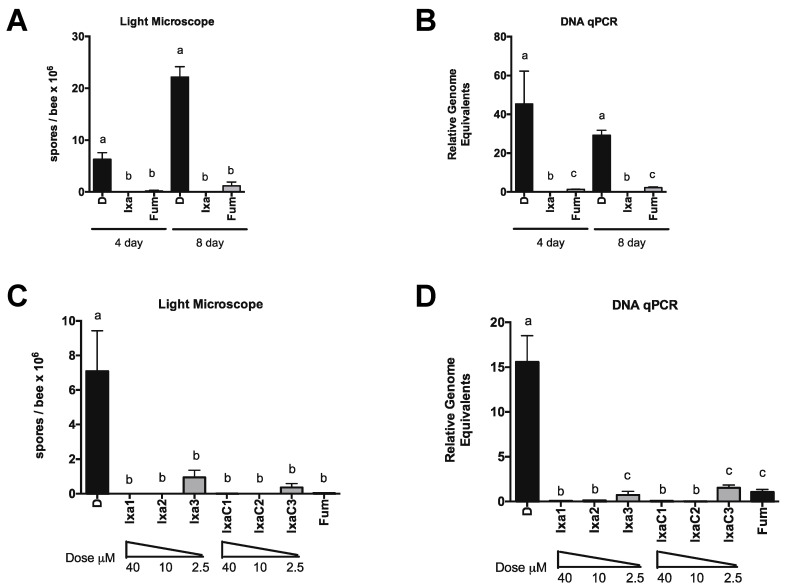
Ixazomib reduces *N. ceranae* infection level in a dose and time dependent manner in newly eclosed bees. (**A**) *N. ceranae* levels in midguts of infected newly eclosed bees fed sucrose solution containing DMSO or ixazomib for 4 or 8 days as determined by spore count using light microscopy (**A**) or by qPCR (**B**) a ≠ b, *p* < 0.05. *N. ceranae* levels in midguts of infected newly eclosed bees fed sucrose solution containing DMSO or various doses of ixazomib for 4 days as determined by spore count using light microscopy (**C**) or by qPCR (**D**) a ≠ b ≠ c, *p* < 0.05.

We focused on ixazomib and ixazomib citrate for further experiments. Again using newly eclosed bees, we treated infected bees for up to 8 days with sucrose solution containing vehicle DMSO, 40 µM ixazomib, or 40 µM fumagillin and measured infection level by spore counting and DNA on days 4 and 8 post initiation of treatment (Figure 4). We also looked at the dose responsiveness of ixazomib and ixazomib citrate on *N. ceranae* infection levels and observed a similar reduction in infection intensity at 10 µM and a diminished reduction in infection intensity at 2.5 µM of both ixazomib and ixazomib citrate by spore counting and DNA analysis (Figure 4C,D). Longer treatment periods did not result in greater decreases in *N. ceranae* infection intensity at doses <10 µM (data not shown).

**Figure 5:**
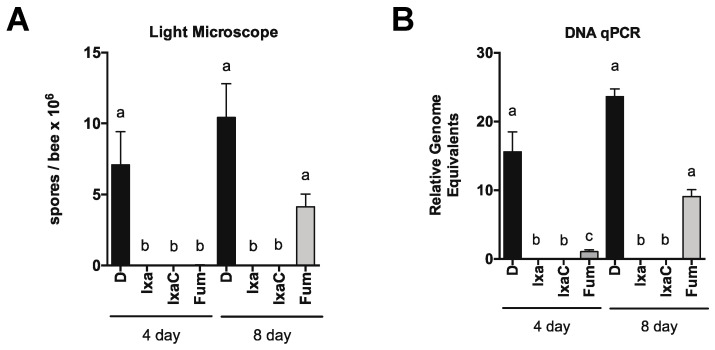
Ixazomib stably reduces *N. ceranae* infection in newly eclosed bees. *N. ceranae* levels in midguts of infected newly eclosed bees fed sucrose solution containing DMSO or ixazomib for 4 days before switching to DMSO alone, as determined by spore count using light microscopy (**A**) or by qPCR (**B**) a ≠ b, *p* < 0.05.

Finally, we wished to determine whether any rebound of *N. ceranae* infection was observed after cessation of treatment by ixazomib and ixazomib citrate as has been reported for fumagillin [18]. We treated infected newly eclosed bees with either sucrose solution containing ixazomib, ixazomib citrate, fumagillin, or vehicle alone for 4 days. We then switched all cages to sucrose solution alone for 4 days and then measured infection level by spore counting and DNA. We observed that infection intensity stayed. The same for bees receiving sucrose solution for the whole experiment, increased for those bees treated with fumagillin first, and decreased for those bees fed either ixazomib or ixazomib citrate first (Figure 5A,B). This suggested that even with a short treatment course, ixazomib and ixazomib citrate can eliminate infection with no evidence of subsequent reemergence. We found that the food consumed by newly eclosed bees did not differ by treatment and on the first day of drug feeding was 26.8 ± 4 μL per bee for 24 h. For 40 μM ixazomib (MW 361.03), this equals 0.39 μg consumed per day. If an adult honey bee is assumed to have a weight of 120 mg [88], then this results in an average of 0.39 mg/0.120 kg or 3.25 mg/kg per day for ixazomib.

### 3.3. Honey Bee Survival Was Unaffected at Doses Up to 40-Fold Those Effective at Reducing N. ceranae Infection Intensity

**Figure 6:**
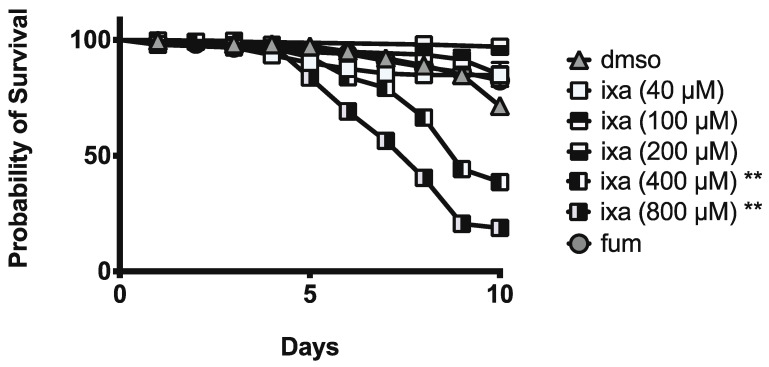
Ixazomib reduces honey bee survival at doses ≥ 40-fold above those that are effective at reducing *N. ceranae* infection intensity. Survival of individual honey bees fed sucrose solution (*n* = 368), fumagillin (40 μM, *n* = 366), or ixazomib (40 μM (*n* = 152), 100 μM (*n* = 47), 200 μM (*n* = 101), 400 μM (*n* = 391), 800 μM ixazomib (*n* = 282)). ** reduced survival relative to control with *p* < 0.01.

To assess the impacts of ixazomib treatment on age-matched honey bees, newly emerged bees were fed sucrose solution containing ixazomib (40, 100, 200, 400, or 800 µM), fumagillin (at 40 µM), or vehicle alone for 10 days starting on 4 days post-eclosion. We found no deceased mortality of bees at 40, 100, or 200 µM of ixazomib. However, we observed decreased survival at 400 and 800 µM doses of ixazomib compared to bees fed sucrose solution containing vehicle alone (Figure 6).

## 4. Discussion

Using a phylogenetic approach, we noted that the *N. ceranae* proteasome appears to lack multiple components observed in most eukaryotes, including multiple free-living fungal species. Examining the RP, we found that *N. ceranae* (and all microsporidia examined) are missing 4 of the 13 genes encoding Rpn subunits (*Rpn3*, *Rpn12*, *Rpn13*, and *Rpn15/Sem1*), and appear to lack at least one of the three known ubiquitin receptors on the proteasome, suggesting a simplified substrate recognition and/or processing mechanism. By contrast, we found that all microsporidia species examined have at least 7 CP α subunits and 7 CP β subunits consistent with all other eukaryotes studied to date. This suggests that the CP is formed from two heteroheptameric α and two heteroheptameric β rings as is the case for other eukaryotes. Genes encoding proteasome assembly chaperones were not detected in *N. ceranae* or other microsporidia. It is important to note that microsporidia proteins are often extremely degenerate relative to their fungal counterparts and it is always formally possible that homologs were missed. *E. cuniculi* was originally thought to lack a gene for Sec61β, a component of the Sec translocon, but a highly divergent Sec61β gene was later discovered in microsporidia [89,90]. Although such chaperones may have gone undetected due to low sequence homology [72], it is also possible that the proteasomes in these organisms have evolved to assemble independent of exogenous chaperone activity. A detailed characterization of the composition and assembly of the microsporidian proteasome will be necessary to support this hypothesis.

We also found a striking reduction in *N. ceranae* infection intensity after pharmacological proteasome inhibition, as measured by spore counts and qPCR. Whereas all proteasome inhibitors had an effect, we found that ixazomib and its citrate salt had the largest effect on pathogen load. Different proteasome inhibitors have very different structures and modes of action within the proteasome [85,86,87]. Ixazomib is a modified peptide boronic acid that works by binding the catalytic site of the β5 subunit of the 20 S proteasome [82]. Ixazomib citrate (MLN9708), which is rapidly hydrolyzed under physiological conditions to its biologically active form, ixazomib (MLN2238), was the first orally bioavailable proteasome inhibitor, originally evaluated for the treatment of multiple myeloma [84]. Ixazomib citrate has demonstrated antitumor activity in a range of tumor xenograft models, as well as multiple myeloma models, and is now an FDA approved drug for multiple myeloma [91].

Targeting the UPS via pharmacologic inhibition of the proteasome [85,86,87] has now become a highly valuable strategy for treating numerous diseases. Of particular interest here, inhibition of proteasome function has been pursued in the treatment of infectious pathogens [92]. The protein degradation machinery is a valuable drug target in the context of other eukaryotic parasites, especially when it is possible to selectively target parasite proteasomes while sparing host proteasomes. The first proteasome inhibitors that were designed to preferentially inhibit eukaryotic pathogen proteasomes while sparing the host were discovered for malaria-causing *Plasmodium* spp. [93,94] and then the kinetoplastid parasites responsible for leishmaniasis, Chagas disease, and sleeping sickness [95]. Such a strategy could be highly valuable for *N. ceranae* treatment. Future studies that further characterize the structural and functional attributes of *N. ceranae* and other microsporidian proteasomes relative to the honey bee host proteasome could provide the basis for the pursuit of rational drug design to discover new proteasome inhibitors or modify existing inhibitors to develop novel compounds that optimize the efficacy to toxicity ratio.

There are a number of reasons why proteasome inhibition might be more toxic for the *N. ceranae* than the honey bee host. One possibility is that the proteasome inhibitors used in this study can preferentially inhibit the *N. ceranae* proteasome relative to that of the honey bee host. Alignment of the β5 subunit homologs from *N. ceranae* and select other species (including the honey bee) show high conservation of the critical ixazomib binding sites, suggesting that any selective inhibition does not occur via differences in this subunit. However, due to missing components (see above), the *N. ceranae* proteasome may have different functional attributes that make it more sensitive to inhibition relative to other eukaryotes. Further work will be required to examine this possibility.

Another reason may be a higher dependence on proteasome function in *N. ceranae* relative to the honey bee host. Microsporidia appear to have a high error rate in protein synthesis, with a high degree of amino acid substitutions in part due to changes in aminoacyl-tRNA synthetase structure [96]. This property may be compounded by the compacted ribosome found in microsporidia that is hypothesized to have reduced quality control function [97,98]. Whereas mistranslation may provide some benefits, such as functional diversity in proteins [96], it may come with costs such as a higher relative load of misfolded proteins that require degradation. We have hypothesized that gene loss during genome compaction has led to reduced redundancy and flexibility to withstand cellular stresses [33]. A clear example of gene loss that would impact proteasome dependence occurs with autophagy, which represents another process involved in the clearance and degradation of proteins [99] with some overlap in function with the proteasome [36]. In other systems, proteasome inhibition often upregulates autophagy function [36], suggesting a compensatory role. Comparative genomics has revealed that microsporidia, like some protozoan parasites, have lost key molecular machinery involved in this process [100]. Thus, inability to rely on autophagy for protein degradation in the absence of proteasome inhibition likely makes this pharmacological disruption especially toxic to microsporidia cells.

Here, we hypothesize that proteasome inhibitors affect microsporidian cells directly by disruption of their proteasomes. It is also possible that the proteasome inhibitors impact the microsporidia indirectly through effects on the honey bee host proteasome [101]. For example, proteasome inhibition could induce a response in these cells that actively reduces *N. ceranae* infection. Two pieces of data argue against this possibility. First, proteasome inhibition has been shown to induce multiple cellular changes, including compensatory gene expression, through a proteasome regulatory network in other metazoans (reviewed in [102]). For example, in *D. melanogaster*, the transcription of select proteasome component genes is increased after genetic disruption of the proteasome [103]. We have shown that proteasome inhibition induces similar gene expression changes in the honey bee and these gene expression changes only occur at doses above those necessary for microsporidia reduction (manuscript in preparation). Second, the survival of the bees for up to 10 days at drug concentrations that are very toxic to *N. ceranae* suggests that proteasome function is not impacted to a dangerous level in the host at these doses. An additional potential mechanism for indirect effects involves the connection between the proteasome and anti-microsporidia immune pathways. One group has found that components responsible for ubiquitination, the proteasome, and autophagy are all important for defense against *N. parisii* infection in *C. elegans* [104]. However, proteasome inhibition would potentially allow for increased *N. ceranae* growth (instead of the observed decrease) if inhibition of the honey bee proteasome was the dominant effect.

When considering therapeutic strategies, it is also important to consider potential negative consequences of proteasome inhibition on honey bee health [101]. We observe that honey bees can tolerate doses of ixazomib up to levels ~40-fold above the concentration necessary to dramatically reduce *N. ceranae* infection (10 μM). This striking resistance to the proteotoxic stress caused by proteasome inhibition in honey bees might be expected. Due to their particular lifestyle, honey bees are exposed to significant routine thermal stress suggesting that the HSR might have unique properties in these insects. Colony-level homeostatic regulation of hive temperature is well recognized as an important adaptive feature of honey bees [105,106], which is maintained by complex individual behaviors, including endothermic shivering to increase heat ([107] and references therein). In maintaining this narrow range of hive temperature and in performing other specialized tasks such as foraging, the temperature of individual bees can increase significantly above steady-state to levels that would be dangerous to other organisms. For example, the temperatures of individual forager bees can reach up to 49 °C in flight [108]. However, honey bees appear highly resistant to thermal stress (reviewed in [109]), and possess a robust heat-shock response [110]. Such resilience may mean they have exceptional systems to combat the disruption in protein homeostasis caused by proteasome inhibitors. However, the impacts of ixazomib and ixazomib citrate (or other inhibitors described here) on individuals of different life stages and castes is not unknown. In particular, the impact on immature stages of bees, such as larvae and pupae will require close scrutiny. Studies in bees show that 20S proteasome activity decreases with aging in honey bee workers [111] although this decrease is not observed in queen bees [112]. Thus, the capacity of bees to withstand proteasome inhibition may differ with age. Perhaps even more obscure is the impact of proteasome inhibitors on overall colony health. Thus, a more comprehensive analysis of the doses and long-term effects of such a treatment strategy on honey bee health, garnered through rigorous field trials, is imperative before they can be used in the management of *Nosema* infection [19].

In a related point, acquired resistance to any therapy is of concern. A recent study showed that *N. ceranae* infection loads eclipsed pre-treatment levels after cessation of fumagillin treatment through an unknown mechanism [18]. Our studies here in *N. ceranae* indicate that no rebound occurs after ixazomib treatment. However, our treatment period is short and we did not examine long-term outcomes at lower doses. Development of resistance in cancers treated with proteasome inhibitors occurs with some frequency. This is typically due to either mutations that disrupt drug binding to the proteasome or overexpression of catalytic β subunits that act as drug sinks [113], although other mechanisms are also possible [114]. Resistance to proteasome inhibitors has also been reported in treatment of infectious disease. For example, malaria parasites develop resistance to non-specific [115] and *Plasmodium*-specific [116] proteasome inhibitors through mutations that impact inhibitor binding. Thus, future studies to examine the stability of suppression and possible routes to resistance in *N. ceranae* should also be undertaken.

*N. ceranae* infection in honey bees can cause individual mortality and contribute to colony collapse. Infection can be treated with fumagillin, but due to concerns about efficacy, toxicity, and future availability, alternative therapeutic strategies are critical. *N. ceranae*, with a highly reduced genome, possesses an atypical proteasome lacking multiple components. We found that pharmacological proteasome inhibition led to a dramatic reduction in *N. ceranae* infection intensity without significant honey bee toxicity. These results suggest that proteasome inhibition can reduce microsporidia infection with limited toxicity to the honey bee host. While rigorous field trials will be required to assess the long-term effects of such a treatment strategy on honey bee health at the individual and colony level, our results offer a convincing novel treatment strategy for microsporidia infection in honey bees.

## Data Availability

Not applicable.

## Supplementary Materials

The following are available online at https://www.mdpi.com/article/10.3390/biom11111600/s1, Figure S1: Comparisons of key Rpn1 and Rpn2 structural domains, Figure S2: *N. ceranae* α ring predictions, Figure S3: Sequence alignment of the 20S proteasome subunit β5 subunit from available microsporidia genomes, Figure S4: Schematic of *N. ceranae* infection of newly eclosed bees, Figure S5: Diverse proteasome inhibitors reduce *N. ceranae* infection in newly eclosed bees, Table S1: Predicted regulatory particle (RP) components, Table S2: Predicted Core Particle (CP) components, Table S3.: Summary of N. ceranae subunit sequence motifs predicted to mediate RP-CP interaction, Table S4: Predicted proteasome assembly components, Table S5: Commercially available proteasome inhibitors used in this study. Predicted proteasome assembly components, Table S6: Comparison of *N. ceranae* reduction versus control, fumagillin, and ixazomib. Video S1: Structural conservation of the *N. ceranae* proteasome.
