# Supplementary material for: Proteasome Inhibition Is an Effective Treatment Strategy for Microsporidia Infection in Honey Bees
Source: Biomolecules. 2021 Oct 29;11(11):1600. doi: 10.3390/biom11111600 (PMC8615682; doi:10.3390/biom11111600)
Supplement: Supplementary file 1 [file biomolecules-11-01600-s001.zip › Supplemental Figures-final.pdf]

Supplemental Figure Legends

A

```
T1 ubiquitin binding site
                                                  *      *   *
hsRPN1      495 EDVLTLLLPVMGDSKSSMEVAGVTALACGMIAVGSCNGDVTSTILQTIMEKSETELKDTY
scRpn1      503 DEVLGLLLPIAASTDLPIETAAMASLALAHVFVGTCNGDITTSIMDNFLERTAIELKTDW
ncRpn1      429 EDLKELLCPLVYSD--NLELSSFAAFTLGSIFVGTGDDEILSVLLQLYIEKED-YAGTHS
ehRpn1      426 EEVRDLLQPLMFSD--SNEVVFFTAFTLGSVFCGSADEDLTSLMLQTFVEKEK-ESETQE
Ub contacts      ^^^^ ^  ^^ ^   ^                    ^ ^^  ^^   ^^

hsRPN1      555 ARWLPLGLGLNHLGKGEAIEAILAALEVVSEPFRSFANTLVDVCAYAGSGNVLKVQQLLH
scRpn1      563 VRFLALALGILYMGQGEQVDDVLETISAIEHPMTSAIEVLVGSCAYTGTGDVLLIQDLLH
ncRpn1      486 YKLIMLGLALLFYRRPD-LECILENMDM---TYSRHGIILIKGFQNIKTGDHNVVEEILT
ehRpn1      483 FRFLMLGLACLFYRRKD-VECGIMEIGG---TLSKHESILIRGFQYVGTGDSNIIESILT
Ub contacts                               ^  ^^  ^^


NT ubiquitin binding site

hsRPN1     272 LNDMELVEDIFTSCKDVVVQKQMAFMLGRHGVFLELSEDVEEYEDLTE--IMSNVQLNSN
scRpn1     288 LGEEDMIRSVFDATSDPVMHKQLAYILAAQKTSF-------EYEGVQD--IIGNGKLSEH
ncRpn1     208 GGYYEDAIDFVGKCEDKKVKKQLLYILARCNIFY-------DTKDLEEKAILCNGHIKDT
ehRpn1     210 RQRIGEAIEYVRGIEDKDYKEQCLYILARCDLYY-------ETSDPNERYILSNGYVKDV
Ub contacts    ^^^^^^^^^^^^^^^^^^^^^^^^^^^^^^^^^^       ^^^
```

B

```
hsRpn2  851 EEKMEVDEAEKKEEKEKKKEPEPNFQ--------LLDNPARVMPAQLKVLTMPETCRYQP
scRpn2  833 EEKEKERETNKKGIKETKENDEEFYKNKYSSKPYKVDNMTRILPQQSRYISFIKDDRFVP
ncRpn2  725 KADENIQSGKRCTLLQMKECGIEFPGVFFTKK----------------------------
ehRpn2  753 ESPSVLTSGSRCTIKQREECGLLSPAIFFVKKK---------------------------


hsRpn2  903 FKPLS-IGGIIILKDTSEDIEELVEPVAAHGPKIEEEEQEPEPPEPFEYIDD------
scRpn2  893 VRKFKGNNGVVVLRD-----REPKEPVALIETVRQMKDVNAPLPTPFKVDDNVDFPSA
ncRpn2      ----------------------------------------------------------
ehRpn2      ----------------------------------------------------------
Rpn13 contacts                                    ^^^^^^^^^^^^^
```

Figure S1. Comparisons of key Rpn1 and Rpn2 structural domains**.** (A) Sequence alignment of portions of the human, *S. cerevisiae*, *N. ceranae*, and *E. hellem* RPN1 orthologs surrounding reported ubiquitin binding sites T1 (top) and NT (bottom) [1,2]. ^, residues in *S. cerevisiae* Rpn1 reported to comprise the ubiquitin binding site. *, residues mutated in the ubiquitin binding-defective *S. cerevisiae* Rpn1 mutant *rpn1-ARR*. (B) Alignment of the C-termini of RPN2 orthologs as in (A). ^, residues in human RPN2 that bind RPN13.

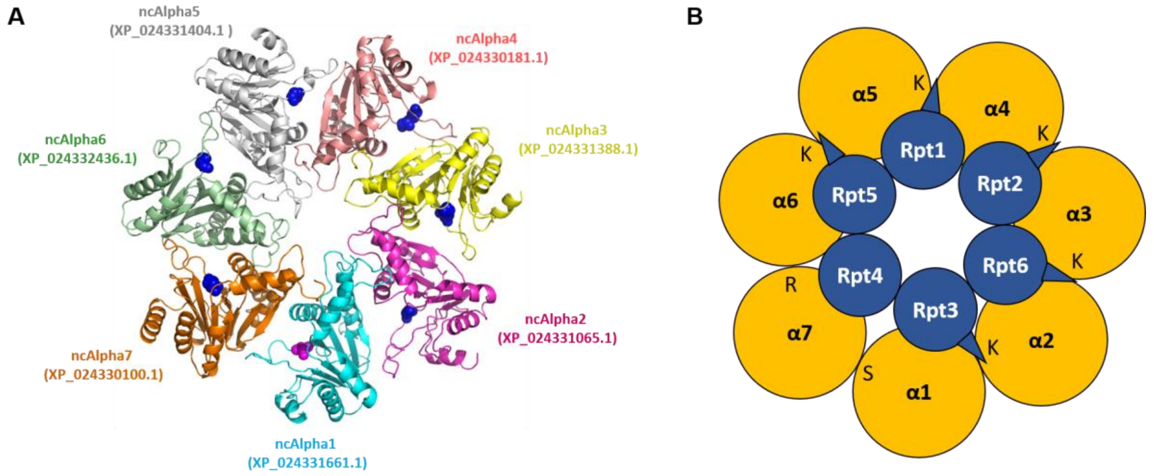

Figure S2. *N. ceranae* α ring predictions. (**A**) Homology model of the *N. ceranae* α ring and hypothesized subunit assignments. Pocket lysines (α2-α6) and arginines (α7) are shown as blue spheres, and the α1 serine 61 in the equivalent position is shown as magenta spheres. (**B**) Cartoon model showing the anticipated register of the RP Rpt subunits on the α ring. The pocket amino acid contributed to a given α-α interface by each α subunit is shown. Rpt subunits contributing Hb-Y-X or Hb-Y-X-like motifs are shown with triangular tails pointing into their respective α subunit pockets.

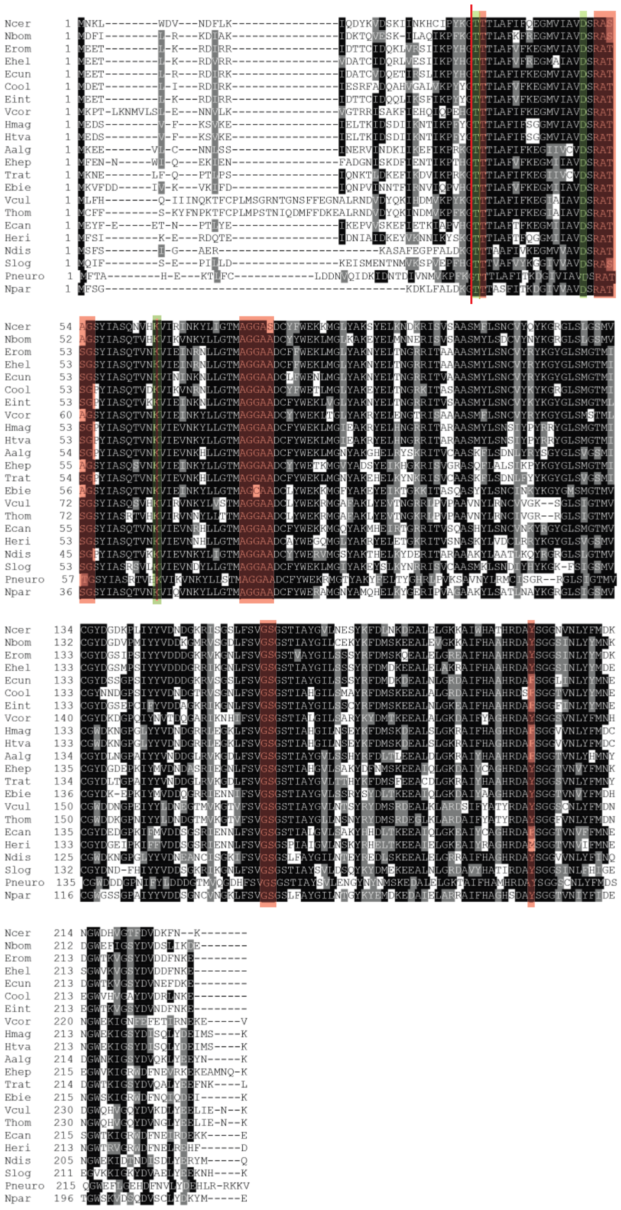

Figure S3. Sequence alignment of the 20S proteasome subunit β5 subunit from available microsporidia genomes. Amino acids from the catalytic triad (T1, D17, and K33 numbered from processed *H. sapiens* protein [3]) are boxed in green and amino acids critical for binding proteasome inhibitors (T2, R19, A20, T21, A22, G23, K33, A46, G47, G48, A49, A50, G129, S130, Y169, numbered from processed *H. sapiens* protein [4]) are boxed in red. The processing site for generating the mature protein is denoted with a red line.

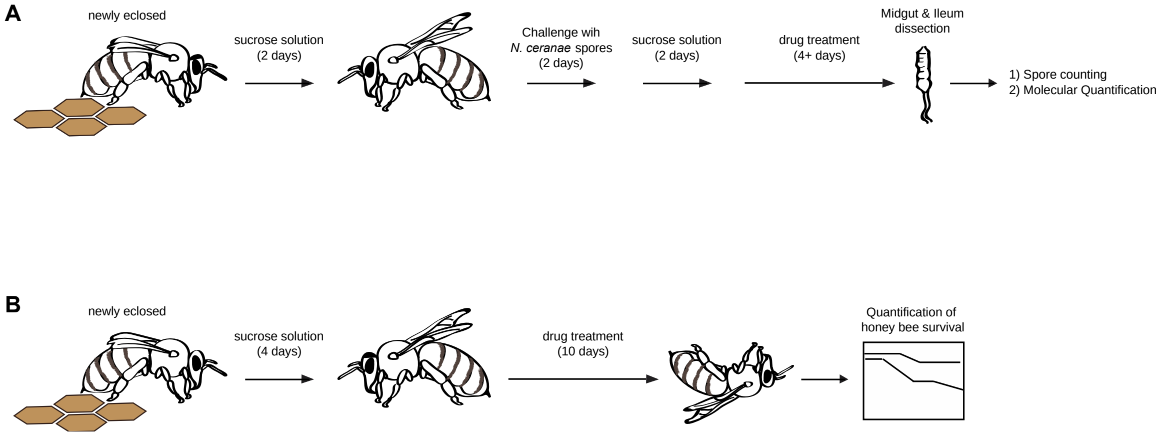

Figure S4. Schematic of *N. ceranae* infection of newly eclosed bees. On day two post-eclosion, *N. ceranae* spores ($5x10^6$ / mL) were fed to bees in sucrose solution *ad libitum* for 48 hours. At 4 days post-infection, honey bees in individual cages were fed sucrose solution containing one of the pharmacologic agents at the indicated doses or vehicle control alone. After 4 days of drug feeding, honey bee midguts were dissected, and infection levels were assessed by spores counting and qPCR. (A) Schematic of survival experiments with newly eclosed bees. At 4 days post-eclosion, honey bees in individual cages were fed sucrose solution containing one of the pharmacologic agents at the indicated doses or vehicle control alone (B).

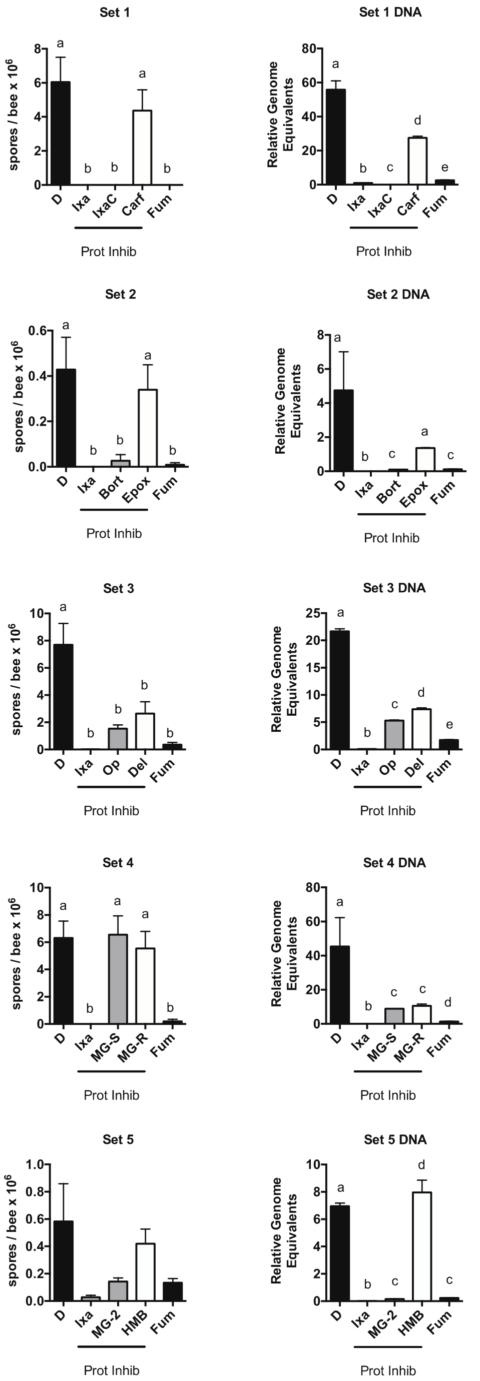

Figure S5. Diverse proteasome inhibitors reduce *N. ceranae* infection in newly eclosed bees. *N. ceranae* levels in midguts of infected newly eclosed bees fed sucrose solution containing DMSO or the indicated compounds for 4 days as determined by spore count using light microscopy (left) or by qPCR (right) a ≠ b, $p < 0.05$.

References

1. Shi, Y.; Chen, X.; Elsasser, S.; Stocks, B. B.; Tian, G.; Lee, B.-H.; Shi, Y.; Zhang, N.; de Poot, S. A. H.; Tuebing, F.; Sun, S.; Vannoy, J.; Tarasov, S. G.; Engen, J. R.; Finley, D.; Walters, K. J. Rpn1 provides adjacent receptor sites for substrate binding and deubiquitination by the proteasome. *Science* **2016**, *351*, aad9421–aad9421.
2. Boughton, A. J.; Liu, L.; Lavy, T.; Kleifeld, O.; Fushman, D. A novel recognition site for polyubiquitin and ubiquitin-like signals in an unexpected region of proteasomal subunit Rpn1. *J Biol Chem* **2021**, *297*, 101052.
3. Huber, E. M.; Heinemeyer, W.; Li, X.; Arendt, C. S.; Hochstrasser, M.; Groll, M. A unified mechanism for proteolysis and autocatalytic activation in the 20S proteasome. *Nature Communications* **2016**, *7*, 10900–10.

4. Schrader, J.; Henneberg, F.; Mata, R. A.; Tittmann, K.; Schneider, T. R.; Stark, H.; Bourenkov, G.; Chari, A. The inhibition mechanism of human 20S proteasomes enables next-generation inhibitor design. *Science* **2016**, *353*, 594–598.
